# Analysis of influencing factors and nursing interventions of patients with mental abnormality after orthotopic liver transplantation

**DOI:** 10.3389/fsurg.2025.1650091

**Published:** 2025-10-23

**Authors:** Jinghui Yan, Li Wu, Yingying Wang, Ruisi Ma, Yanmin Shi, Yajie Chen

**Affiliations:** Department of Liver and Gall Surgical, The Third Hospital of Hebei Medical University, Shijiazhuang, Hebei, China

**Keywords:** orthotopic liver transplantation, mental abnormality, influencing factors, nursing, interventions

## Abstract

**Objective:**

To analyze the influencing factors and nursing interventions of patients with mental abnormality after orthotopic liver transplantation.

**Methods:**

The clinical data of one hundred patients received orthotopic liver transplantation in our hospital from January 2022 to June 2023 were selected to be study objects. Patients were divided into research group (40 cases) and control group (60 cases) according to whether psychiatric symptoms occurred after surgery. Correlation statistical analysis methods such as univariate Logistic regression and binary logistic regression were used to analyze the correlation between the general condition, preoperative, intraoperative and postoperative related indicators and the occurrence of postoperative neuropsychiatric complications in the two groups.

**Results:**

There were no deaths during perioperative period. Neuropsychiatric complications occurred in 40 of 100 cases. Univariate analysis results displayed that there were significant differences in preoperative hepatic encephalopathy and grade C liver function between 2 groups (*P* < 0.01), there was significant difference in the average amount of intraoperative blood loss between the two groups (*P* < 0.01), and there were significant differences in postoperative intensive care unit (ICU) treatment time, FK506 concentration, and electrolyte disturbance between 2 groups (*P* < 0.05 and *P* < 0.01). Binary Logistic regression analysis results showed that grade C liver function, postoperative ICU treatment time, FK506 concentration and electrolyte disturbance were risk factors for neuropsychiatric complications after liver transplantation (*P* < 0.01).

**Conclusion:**

Grade C liver function, postoperative ICU treatment time, FK506 concentration, and electrolyte disturbance are risk factors for neuropsychiatric complications after liver transplantation. According to the above risk factors, standardized management and psychological counseling should be carried out.

## Introduction

With the improvement of medical level, especially the rapid development of surgical techniques, liver transplantation surgeries have been widely used in clinical practice (1). Liver transplantation is a highly complex surgical procedure that requires a team effort, and many complications may occur after the operation (2). Neuropsychiatric complications are one of the common complications after liver transplantation (3). According to a comprehensive international multicenter study, the overall incidence of neuropsychiatric complications after liver transplantation ranges from 15% to 40% (4). According to the DSM-5 criteria and clinical studies, postoperative mental disorders mainly manifest in three categories: organic mental disorders, anxiety/depression disorders, and post-traumatic stress disorder (PTSD) (5). These neuropsychiatric complications will lead to autonomic nerve dysfunction, increases myocardial oxygen consumption, and induces arrhythmias (6). Meanwhile, these complications will inhibit immune cell function, increases the risk of infection, prolong the hospital stay of patients and increase medical costs (7). In addition, most of the neuropsychiatric complications after liver transplantation occur within 1 month after the operation. The core mechanism is direct damage to the central nervous system, which not only brings difficulties to clinical treatment, but also directly affects the treatment outcome and quality of life of patients, and may even lead to an increase in perioperative mortality (8). Although the number of reports and studies on this complication has been increasing in recent years, the current research on its occurrence and development mechanism is still not very clear (9). Some studies have shown that the occurrence and development of neuropsychiatric complications is related to immune suppressant neurotoxicity, metabolic disorder synergy, and multi-organ axis imbalance (10). Moreover, due to the lack of specific clinical manifestations, early diagnosis is extremely difficult, and there are no targeted treatment methods available. This has led to an increase in the perioperative mortality rate of patients after surgery (11). Therefore, conducting in-depth research on the relevant factors of neuropsychiatric complications after liver transplantation, and implementing targeted nursing intervention measures for these situations, is of great significance for clinical prevention and treatment of mental symptoms.

Herein, our study aimed to explore the influencing factors and nursing interventions of patients with mental abnormality after orthotopic liver transplantation.

## Data and methods

### General data

The clinical data of one hundred patients received orthotopic liver transplantation in our hospital from January 2022 to June 2023 were selected to be study objects. There were 60 males and 40 females. They ranged in age from 30 to 70, with a mean age of (49.85 ± 6.58) years. The primary diseases were 58 cases of posthepatitic cirrhosis, 16 cases of alcoholic cirrhosis, and 26 cases of primary liver cancer. All the above results were confirmed by histopathological examination. A total of 40 patients with neuropsychiatric complications were selected as the research group, and 60 patients with no neuropsychiatric complications served as the control group. The definition of neuropsychiatric complications was based on the 11th edition of the International Classification of Diseases (ICD-11) and the Diagnostic and Statistical Manual of Mental Disorders (Fifth Edition) (DSM-5). All patients underwent standardized neuro-psychiatric assessments every day after the surgery, which were conducted jointly by nurses and doctors. The nurse used the Richmond Agitation-Sedation Scale (RASS) to monitor the patient's level of consciousness. When the RASS score is ≤ −3 or ≥ 2, a psychiatric consultation was triggered. A structured clinical interview was conducted by a psychiatrist with experience in liver transplantation (with an average working experience of 8 years). Based on the DSM-5 criteria and auxiliary examinations, the final diagnosis was made. Data collection was accomplished through electronic case report forms (eCRF), including baseline mental health status and postoperative neuropsychiatric events. One week before the surgery, the anesthesiology doctor conducted a simple mental state examination (MMSE, with a score of ≥24 indicating normal cognition) and the HADS scale (with a score of ≤7 indicating no anxiety/depression) to screen the patient's mental health status. An independent data administrator randomly selected 10% of the cases and verified the consistency between the original medical records and the eCRF. To ensure that there was no history of mental illness before the surgery, a multi-source information cross-validation method was adopted, including electronic medical record review, family interviews, and the preoperative Patient Health Questionnaire-9 (PHQ-9) screening. Patients with PHQ-9 scores ≥10 points were excluded. There were no significant differences in age, gender and primary disease type between the two groups (*P* > 0.05, Table 1).

**Table 1:** General data of patients in 2 groups.

Items	Control group (*n* = 60)	Research group (*n* = 40)	t/*χ*^2^	P
Gender (male/female)	49.06 ± 6.42	50.13 ± 6.58	0.80	>0.05
Age (years)	35/25	25/15	0.17	>0.05
Primary disease type				
Posthepatitic cirrhosis	36	22	0.80	>0.05
Primary liver cancer	16	10		
Alcoholic cirrhosis	8	8		

### Data collection

Preoperative data: Both donor and recipient were in line with the ABO blood group matching principle, and the donor liver was in good health. Gender, age, complication of hepatic encephalopathy and preoperative liver function level were collected.

Intraoperative data: The operative time, intraoperative blood loss, anhepatic period and donor cold ischemia time were collected.

Postoperative data: Intensive care unit (ICU) treatment time, serum FK506 concentration, albumin level, obvious infection, electrolyte disturbance, and pathological diagnosis. After liver transplantation, tacrolimus (FK506) + mycophenolate motiate (Cellcept) + methylprednisolone triple anti-rejection therapy was routinely used. The initial dose of tacrolimus was 0.05 mg/kg, which was injected through a gastric tube every 12 h and changed to oral administration after gastrointestinal function was recovered. Blood concentration, blood routine, blood liver biochemistry, and kidney function were monitored after surgery, and the dosage was adjusted according to blood concentration and hematology results. The FK506 valley concentration was maintained at 8–13 ng/ml within 0–3 months after surgery, and at 7–10 ng/ml within 3–6 months after surgery. The valley concentration of FK506 was maintained at 6–8 ng/ml 6–12 months after surgery, and the valley concentration of long-term survival maintenance dose was 3–5 ng/ml after surgery. Mycophenolate morpheate was 0.5 g/d, once every 12 h. Blood routine, liver and kidney function were monitored. Methylprednisolone was injected intravenously, the hormone was gradually removed after surgery, and low dose oral maintenance therapy was performed. For cancer patients, hormone withdrawal was carried out as quickly as possible, and any side effects caused by hormone use were monitored.

### Statistical analysis

The correlation between various factors and postoperative psychiatric symptoms was analyzed. Statistical analysis software SPSS 20.0 was used for statistical analysis of the survey data. First, a single factor analysis was carried out to evaluate these related factors. The measurement data conforming to normal distribution were represented by mean ± standard deviation, and the comparison between the two groups was performed by *t*-test. Statistical data were compared using *χ*^2^ test. Then, the statistically significant factors in the univariate analysis were included in the binary Logistic regression analysis to further determine the risk factors for neuropsychiatric complications after liver transplantation. *P* < 0.05 was considered statistically significant.

## Results

### Occurrence of postoperative neuropsychiatric complications

Neuropsychiatric complications occurred in 40 of 100 cases. There were 14 cases of agitation, 12 cases of anxiety with insomnia, 7 cases of hallucination, 5 cases of visual disturbance, and 2 cases of limb movement disorder, as displayed in Table 2.

**Table 2:** Analysis of clinical manifestations of mental symptoms in research group.

Symptom type	Cases	Percentage	Incidence rate
Agitation	14	35.00%	14.00%
Anxiety with insomnia	12	30.00%	12.00%
Hallucination	7	17.50%	7.00%
Visual disturbance	5	12.50%	5.00%
Limb movement disorder	2	5.00%	2.00%

### Univariate analysis of patients’ general condition and preoperative clinical data

There were significant differences in hepatic encephalopathy and grade C liver function between 2 groups (*P* < 0.05), as displayed in Table 3.

**Table 3:** Univariate analysis of patients’ general condition and preoperative clinical data.

Items	Control group (*n* = 60)	Research group (*n* = 40)	t/χ^2^	P
Age (years)	49.06 ± 6.42	50.13 ± 6.58	0.80	>0.05
Gender (male/female)	35/25	25/15	0.17	>0.05
Hepatic encephalopathy (Yes/No)	15/45	30/10	24.24	<0.01
Grade C liver function (Yes/No)	13/47	21/19	10.17	<0.01

### Univariate analysis of intraoperative data in two groups

There was significant difference in the average amount of intraoperative blood loss between the two groups (*P* < 0.01), as revealed in Table 4.

**Table 4:** Univariate analysis of intraoperative data in two groups.

Items	Control group (*n* = 60)	Research group (*n* = 40)	t	P
Operation time (h)	7.23 ± 0.75	7.32 ± 0.76	0.58	>0.05
Intraoperative blood loss (ml)	5,458 ± 546.23	4,012 ± 400.26	15.26	<0.01
Anhepatic duration (min)	47.56 ± 4.75	48.26 ± 4.83	0.71	>0.05
Donor cold ischemia time (h)	4.95 ± 0.52	5.06 ± 0.57	0.98	>0.05

### Univariate analysis of postoperative clinical data of two groups

Table 5 displayed that there were significant differences in postoperative ICU treatment time, FK506 concentration, and electrolyte disturbance between 2 groups (*P* < 0.05 and *P* < 0.01).

**Table 5:** Univariate analysis of postoperative clinical data of two groups.

Items	Control group (*n* = 60)	Research group (*n* = 40)	t/χ^2^	P
Duration of ICU treatment (h)	2.62 ± 0.27	4.59 ± 0.46	24.43	<0.01
FK506 concentration (ng/ml)	9.82 ± 0.10	16.48 ± 1.65	25.50	<0.01
Infection (Yes/No)	25/35	23/17	2.41	>0.05
Electrolyte disturbance (Yes/No)	34/26	32/8	5.82	<0.05
Postoperative albumin level (g/L)	32.52 ± 3.26	32.41 ± 3.21	0.17	>0.05

### Binary logistic regression analysis results

The indexes of hepatic encephalopathy, grade C liver function, intraoperative blood loss, postoperative ICU treatment time, FK506 concentration, and electrolyte disturbance with statistical significance (*P* < 0.05) in the above univariate analysis were included in binary Logistic regression analysis. The results showed that grade C liver function, postoperative ICU treatment time, FK506 concentration and electrolyte disturbance were risk factors for neuropsychiatric complications after liver transplantation (*P* < 0.05, Table 6).

**Table 6:** Binary logistic regression analysis results.

Influencing factors	*β*	Standard error	OR value	95% (CI)	P
Hepatic encephalopathy	1.	1.58	3.16	1.02-9.48	>0.05
Grade C liver function	2.15	0.72	8.39	2.20–32.28	<0.05
Intraoperative blood loss	0.02	0.06	1.02	0.98–1.05	>0.05
Postoperative ICU treatment time	1.82	0.56	11.65	2.18–17.28	<0.05
FK506 concentration	1.62	0.62	6.85	1.53–15.62	<0.05
Electrolyte disturbance	1.52	0.78	5.82	1.12–18.68	<0.05

## Discussion

Our study indicated that grade C liver function, postoperative ICU treatment time, FK506 concentration, and electrolyte disturbance were risk factors for neuropsychiatric complications after liver transplantation. Before undergoing liver transplantation surgery, the liver function of most patients is already poor (12). After the surgery, the liver function of the patients is in the recovery stage, and the metabolic rate of toxic substances in the body slows down, which leads to mental symptoms such as restlessness and auditory hallucinations after the operation (13). After liver transplantation, patients need to be closely observed and treated in the ICU. Long-term postoperative monitoring can seriously disrupt the patients’ daily rhythm and lead to negative psychological pressures such as depression and loneliness (14). The postoperative abdominal drainage tube and intravenous infusion channels prevent patients from changing their body positions independently. In addition, the unique diagnostic and treatment environment in the ICU, such as the sounds emitted by various monitoring instruments, the tense atmosphere beside the bed during rescue, and the high-frequency diagnostic and treatment operations, are all situations that the patients have never experienced before, which can trigger neuro-psychiatric symptoms in liver transplant patients (15). Consistently, Desai et al. suggested that common neuropsychiatric complications, including cognitive impairment and symptoms of depression and posttraumatic stress disorder, are frequently associated with ICU sedation, delirium or delusional memories, and long-term impairments in quality of life (16). Liver is the most important site for drug metabolism and the target organ for drug damage (17). When immunosuppressive therapy is applied, its toxicity and side effects are easily overlooked (18). Recent studies have shown that the use of tacrolimus may cause symptoms such as epilepsy in liver transplant patients (19). The mental disorders caused by immunosuppressants after liver transplantation may be closely related to neurotoxicity, but the specific mechanism remains unclear (20). Some studies have found that immunosuppressants exert their effects by forming complexes with immune-affinity proteins (21). Immune-affinity proteins are widely present in cells of the central nervous system and have a protective effect on neurons of the central nervous system, so this may be one of the mechanisms leading to mental disorders (18, 22). Similarly, among the kidney transplant recipients, long-term use of drugs such as tacrolimus, which belong to the class of calcineurin inhibitors, may cause neurotoxic reactions, leading to mental symptoms such as anxiety and depression in patients (23). This phenomenon may be related to the direct toxic effect of the drug on the nervous system, as well as its interference with the normal metabolism and function of neurotransmitters (24).

Many patients who undergo liver transplantation will experience varying degrees of electrolyte imbalance and acid-base imbalance before the surgery, such as low potassium levels, low sodium levels, high sodium levels, low calcium levels, and low magnesium levels. During the surgery, there will be a large amount of fluid exchange, and after the surgery, there may be inappropriate fluid supplementation, which will lead to water, electrolyte and acid-base balance disorders, which may cause mental symptoms (25).

In addition, effective nursing interventions play an important role in patients with mental abnormality after orthotopic liver transplantation, including (1) Preoperative psychological assessment: Before performing the liver transplantation surgery, nurses should conduct psychological assessments of the patients using HADS scale. When the scores of the anxiety or depression scale are ≥ 8 points, it indicates that the patient may have anxiety or depression, and further assessment and intervention are required. Then, nurses develop personalized psychological care plans based on the patients’ psychological characteristics. Nurses explain the relevant disease knowledge to the patients, introduce the achievements of modern transplantation surgery and the risks of the surgery, so that the patients and their families can fully understand the liver transplantation surgery and accept it in a psychologically adapted state (26). When communicating with patients, nurses should give them sufficient attention. They can do this by making eye contact, nodding, and other gestures to show that they are listening carefully. Nurses should respect the patients’ right to express themselves and should not interrupt the patients’ speech at will. Even if the nurses have different opinions from the patients’ viewpoints, they should first allow the patients to fully express their thoughts, and then communicate and interact with them in a gentle and rational manner. Since transplant patients and their families may have limited knowledge of medical matters, nurses should avoid using overly technical medical terms when communicating with them. Instead, they should explain the condition, treatment plans, and care measures in plain and understandable language. Nurses should understand the feelings of patients from their perspective and express empathy through both verbal and non-verbal means. Nurses can regularly organize health lectures for the family members of patients who have undergone orthotopic liver transplantation, informing them about the patient's condition, treatment plan, post-operative care precautions, and the significance of mental health issues. Nurses prepare some written materials about post-operative care and mental health for the family members, such as brochures and care guidelines. These materials can include manifestations of common psychological problems after the surgery, coping strategies, the role of family support, to facilitate the family members’ easy access and learning. Nurses organize patients’ family members with similar experiences to form a mutual support group. During the group activities, the family members can share their care experiences and feelings, support and encourage each other. Nurses regularly participate in the group activities and provide professional guidance and advice to the family members, helping them better deal with the psychological health problems of the patients. Before the surgery, nurses allow the patients to familiarize themselves with the environment of the transplantation center and learn more about the postoperative monitoring and treatment, so that the patients can make appropriate psychological preparations. (2) Postoperative psychological support: After the surgery is completed, the nurse should promptly inform the patient of the relevant information about the successful operation and provide objective indicators of the survival of the transplanted liver to alleviate the patient's fear (27). The nurse should thoroughly explain the purpose of each catheter's placement and take comfortable nursing measures for various postoperative discomforts. The nurse should enhance communication with the patient, encourage the patient to express their feelings, and adopt supportive psychological treatment methods, such as emotional transmission, positive suggestion, and literary inspiration provided by the nursing staff, to correct the patient's negative emotions, allowing the patient to forget the pain and enter the best state of mind and body. (3) Establishment of a comfortable environment: Nurses should appropriately arrange family visits to meet the patients’ needs for love and a sense of belonging. Nurses allow patients to listen to music and watch TV, to distract their attention, eliminate irritability and fear. Nurses try to reduce the number of unnecessary examinations and treatments as much as possible, and transfer patients out of the ICU as soon as their condition stabilizes. (4) Active treatment of primary disease and complications: Postoperative complications are an important factor affecting the survival rate of liver transplant patients, and they directly impact the quality of life of these patients. Therefore, it is necessary to strengthen postoperative observation (28). Nurses should closely monitor vital signs, the nature, and volume of bile and abdominal drainage fluid, liver and kidney functions, interleukin-2 receptor (IL-2R) and other rejection reaction indicators, and promptly handle abnormal situations. At the same time, nurses should maintain the stability of the internal environment and the normal concentrations of calcium, magnesium, sugar and cholesterol in the blood, and quickly correct abnormal conditions. (5) Pharmaceutical administration: After the surgery, the nurse should administer the prescribed medications to the patient on time and in the correct dosage, closely monitor the blood concentration of the immunosuppressant drugs, carefully observe the side effects of the drugs and the interactions between them, pay attention to the timing and pattern of the occurrence of mental abnormalities, and promptly adjust the dosage and usage time of the medication to minimize the adverse reactions of the drugs (29). (6) Observation and treatment of mental abnormalities: After surgery, nurses should promptly understand the patient's rest state as well as their psychological and emotional changes, closely observe the patient's state of consciousness, abnormal personality and behavior, insomnia, irritability and other symptoms, immediately report to the doctor, promptly carry out treatment and rule out the precursors of hepatic encephalopathy. For patients with abnormal mental states, nurses should try to avoid using restraints to prevent the aggravation of agitated symptoms. Nurses should frequently inquire about the patient's condition, provide psychological counseling, prevent accidental injuries, and meet safety needs. In necessary cases, they can switch to immunosuppressants or stop or reduce some drugs that stimulate the excitation of the nervous system. (7) Discharge guidance: Before the patient is discharged from the hospital, the nurses should explain in detail to the patient and their family members the timing, dosage and possible side effects of taking the immunosuppressants, and ensure that the patient takes the medication on time. After discharge, the nurse should inform the patient to regularly check the drug concentration in the blood and that any drug adjustments should be carried out under the guidance of the doctor. The nurse should also inform the patient that if there are no adverse reactions, they should return to the hospital at any time. The nurse suggests that the patient appropriately engage in less stressful work and maintain a good mental state.

Although this study identified some factors influencing post-operative mental disorders after orthotopic liver transplantation, the underlying biological mechanisms of the disease remain poorly understood. Future research could focus on aspects such as neuroimmunology, neuroendocrinology, and neuroplasticity. In addition, the rehabilitation of liver transplant patients is a long-term process, and the occurrence and development of mental abnormalities may also change over time. In the future, we will conduct long-term follow-up studies to track the mental health status of patients several years after the surgery. This will enable us to have a more comprehensive understanding of the evolution patterns of mental abnormalities, the dynamic changes of influencing factors, and the long-term effects of nursing intervention measures.

## Limitations

Our research has some limitations. Firstly, our sample size is relatively small, which may lead to deviations between the data results and the actual values. Secondly, our research was a single-center study, and the sample was not representative, which may not accurately reflect the characteristics of a broader population. Therefore, more multi-center and large-scale studies should be conducted in the future to further verify our findings.

## Conclusion

Grade C liver function, postoperative ICU treatment time, FK506 concentration, and electrolyte disturbance are risk factors for neuropsychiatric complications after liver transplantation. According to the above risk factors, standardized management and psychological counseling should be carried out.

## Data Availability

The datasets presented in this study can be found in online repositories. The names of the repository/repositories and accession number(s) can be found in the article/Supplementary Material.
